# Supplementary material for: Peri-Interventional Triple Therapy with Dabigatran Modifies Vasomotion after Bare-Metal Stent Implantation in a Pig Coronary Artery Model
Source: J Pers Med. 2023 Jan 31;13(2):280. doi: 10.3390/jpm13020280 (PMC9962101; doi:10.3390/jpm13020280)
Supplement: Supplementary file 1 [file jpm-13-00280-s001.zip › jpm-2146547-supplementary.pdf]

Supplementary Material

Peri-interventional triple therapy with dabigatran modifies vasomotion after bare-metal stent implantation in a pig coronary artery model

Rayyan Hemetsberger, Serdar Farhan, Dominika Lukovic, Katrin Zlabinger, Judit Hajagos-Toth, Judit Bota, Hector M Garcia-Garcia, Cihan Ay, Eslam Samaha, Robert Gaspar, Rita Garamvölgyi, Kurt Huber, Andreas Spannbauer & Mariann Gyöngyösi

Supplementary Material

Histomorphometry and histopathology

Histopathology and histomorphometry assessments were carried out by experienced observers without knowledge of the stent type or medication group. Each stented segment was sectioned into three pieces (proximal, middle [bearing stent], and distal) and evaluated following the recommendations of Schwartz et al. (1).

Briefly, inflammation was graded as follows: 0, no or minimal inflammatory cells in media and adventitia; 1, mild diffuse inflammatory infiltration or moderate focal areas <25% of the vessel media and adventitia; 2, moderate infiltration or marked, focal areas covering 25%–50% of the media and adventitia; 3, heavy infiltration or marked, focal areas >50% of the media and adventitia; and 4, granulomatous inflammatory reaction in any layer of the vessel wall. Fibrin score was graded from 0 to 3, respectively, as follows: no fibrin deposition (0), or fibrin deposition involving <10% (score 1), 10%–25% (2), or >25% (3) of the circumference of the vessel. Injury severity was graded as follows: 0: no injury, with intact internal and external elastic lamina and media; 0.5: minimal disruption of internal lamina, intact external lamina and media; 1: lacerated internal lamina, external lamina and media intact; 1.5: lacerated internal lamina, less-than-half-thickness laceration of media, intact external elastic lamina; 2: lacerated internal elastic lamina, media lacerated more than half thickness, intact external elastic lamina; 2.5: lacerated internal elastic lamina and media (full thickness), minimal disruption of external lamina; 3: all lacerated (media, full thickness).

To calculate endothelial coverage, we took the percentage of the lumen circumference that the endothelium covered, as measured using computerized planimetry (ImageJ version 1.440, NIH, Bethesda, MD).

We quantified the internal and external elastic lamina areas and maximal neointimal thickness of the lumen, along with calculating the neointima area (difference between internal

elastic lamina and lumen area), media area (difference between external and internal elastic lamina area), and % area stenosis [(neointimal area/internal elastic lamina area) x 100].

A matched analysis of in vivo OCT and ex vivo histology could not be performed because no external marker could be implanted to serve as a reference to ensure the precise correlation of individual cross-sections.

References

1. Schwartz RS. Edelman E. Virmani R et al. Drug-eluting stents in preclinical studies: updated consensus recommendations for preclinical evaluation. Circ Cardiovasc Interv 2008;1:143-53.

Supplementary Tables

Supplement Table S1. Quantitative angiographic results immediately after PCI with BMS at day 3 and at one-month follow-ups.

%DS: percent diameter stenosis; FUP: follow-up; LLL: late lumen loss; MLD: minimal lumen diameter; post-PCI: immediately after PCI; RD: reference diameter

Mean ± SD

A. Baseline and day 3 follow-up

	**Baseline RD** (mm)	**Post-PCI MLD** (mm)	**Post-PCI %DS** (%)	**3d FUP RD** (mm)	**3d FUP MLD** (mm)	**3d FUP %DS** (%)	**3d FUP LLL** (mm)
**Dabigatran group**	**2.87** ±0.25	**2.94** ±0.39	**3.00** ±4.80	**3.15** ±0.35	**2.95** ±0.34	**6.54** ±5.38	**-0.01** ±0.32
**Control group**	**2.92** ±0.47	**2.83** ±0.30	**6.25** ±8.01	**3.02** ±0.32	**2.73** ±0.40	**9.92** ±7.97	**0.04** ±0.50
P	0.77	0.40	0.18	0.35	0.15	0.20	0.77

B. One-month follow-up

	**RD** (mm)	**MLD** (mm)	**%DS** (%)	**LLL** (mm)
**Dabigatran group**	**3.25** ±0.53	**2.06** ±0.70	**36.64** ±21.30	**0.80** ±0.54
**Control group**	**3.18** ±0.33	**2.07** ±0.67	**34.04** ±23.18	**0.83** ±0.57

P	0.73	0.97	0.82	0.93

Supplement Table S2. Quantitative OCT results immediately after PCI with BMS at day 3 and at one-month follow-ups.

FUP: follow-up; post-PCI: immediately after PCI

Mean ± SD

	**Post-PCI lumen area** (mm^2)	**3-day FUP lumen area** (mm^2)	**1-month FUP lumen area** (mm^2)	**1-month FUP area stenosis** (%)
**Dabigatran group**	**8.62** ±1.74	**7.81** ±1.26	**4.58** ±1.98	**37.95** ±22.25
**Control group**	**8.36** ±1.19	**8.29** ±1.11	**4.85** ±1.75	**42.98** ±19.11
P	0.74	0.37	0.78	0.65

Supplement Table S3. Histomorphometric results.

1mo: 1-month follow-up; 3d: 3-day follow-up

Mean ± SD

	**Lumen area** (mm^2)		**Intimal area** (mm^2)		**IEL area** (mm^2)		**EEL area** (mm^2)		**Stent stenosis** (%)	
	**3d**	**1mo**	**3d**	**1mo**	**3d**	**1mo**	**3d**	**1mo**	**3d**	**1mo**
**Dabigatran group**	**4.80** ±0.86	**2.26** ±0.97	**0.26** ±0.34	**2.06** ±0.62	**5.05** ±1.00	**4.32** ±0.41	**5.89** ±1.15	**5.93** ±0.74	**5** ±5	**49** ±19
**Control group**	**5.14** ±1.00	**2.75** ±1.38	**0.15** ±0.10	**2.90** ±1.63	**5.29** ±1.01	**5.65** ±0.89	**6.48** ±0.66	**6.71** ±0.95	**3** ±2	**51** ±25
P	0.54	0.55	0.56	0.36	0.70	0.02	0.36	0.19	0.52	0.91
